# The role of stigma in impeding implementation of harm reduction services in San Francisco

**DOI:** 10.1016/j.ssmqr.2025.100593

**Published:** 2025-06-26

**Authors:** Christopher F. Akiba, Cariné E. Megerian, Esther O. Chung, Terry Morris, Lynn D. Wenger, Leslie W. Suen, Barrot H. Lambdin, Alex H. Kral

**Affiliations:** a RTI International, 3040 Cornwallis Rd, Research Triangle Park, NC, 27709, USA; b UCSF Division of General Internal Medicine at San Francisco General Hospital, 1001 Potrero Ave, San Francisco, CA, 94110, USA

**Keywords:** Stigma, Policy, Harm reduction, Implementation

## Abstract

The United States continues to face an epidemic of drug overdose deaths among people who use drugs (PWUD). Harm reduction services are efficacious interventions that reduce overdose deaths and improve the health of PWUD. For decades, San Francisco has remained at the vanguard of the adoption, implementation, and sustainability of harm reduction programs. During a time of national urgency in 2023, we conducted a qualitative study interviewing leaders, managers, and frontline staff at 10 community-based organizations providing harm reduction services in San Francisco. We analyzed in-depth interview data using Reflexive Thematic Analysis. Participants described feeling that PWUD, providers of harm reduction services, and the services themselves became highly stigmatized at the political and community levels. Multi-level stigma was exacerbated by the city’s social and economic conditions including extreme income inequality and gentrification, giving rise to public drug use and open-air drug markets. Multi-level stigma was upheld by a system of anti-harm reduction rhetoric and misinformation from public officials, leading to its politicization and insufficient funding for organizations providing harm reduction services. These barriers resulted in harm reduction worker self-censorship and staff trauma, burnout, and turnover, leading to program implementation challenges and ultimately harming organizations’ abilities to improve the health of PWUD. Organizations mitigated barriers through mutual aid but only to a degree. Targeting stigma directly may help to address implementation challenges over the long term and lead to additional, sufficient, and sustained funding needed to ensure adequate and stable service provision.

## 1. Introduction

The United States (US) continues to face an epidemic of drug overdose deaths among people who use drugs (PWUD), with over 100,000 in 2023 (CDC, 2024). Some of the major health complications associated with drug use include overdoses (Mattson et al., 2021; O’Donnell et al., 2021), viral and bacterial infections (Degenhardt et al., 2023; Larney et al., 2017), co-morbid mental health conditions (McKetin et al., 2019; NIDA, 2020; Sullivan et al., 2006), as well as sores, burns, and cuts (Butler et al., 2017; Fischer et al., 2008). Drug overdoses are one of the leading causes of death for adults in the US, and recent declines in life expectancy have been largely attributed to the overdose mortality epidemic (Spencer et al., 2024; Woolf & Schoomaker, 2019).

A large body of research points to the role that stigma plays in undermining access to and utilization of healthcare services (Carl et al., 2023; Earnshaw, 2020; Hatzenbuehler et al., 2013; Nyblade et al., 2019). The presence of stigma against PWUD from healthcare providers is abundantly detailed in the literature, with negative attitudes leading to suboptimal quality of healthcare (Biancarelli et al., 2019; Miller-Lloyd et al., 2020; Simon et al., 2020; van Boekel et al., 2013) and poorer outcomes (Jaibat et al., 2023).

Many community-based organizations (CBOs) have implemented evidence-based harm reduction interventions, which can facilitate more accessible healthcare through reduced stigma. CBOs that offer harm reduction services represent essential healthcare settings for PWUD because many employ a non-stigmatizing and radically hospitable approach (Brandon Muncan et al., 2020; Wenger et al., 2024). Staff at such organizations are culturally competent in providing services to PWUD and many organizations have delivery systems designed to reach participants wherever they are (Corneli et al., 2022; Muncan et al., 2021). These services include syringe services programs (SSPs), overdose education and naloxone distribution (OEND) programs, distribution of fentanyl test strips (FTS), community-based Fourier-transform infrared spectroscopy drug checking (FTIR), overdose prevention sites (OPS), sobering centers, and overdose detection technologies (Table 1). Despite the documented efficacy of evidence-based harm reduction interventions, CBOs that provide such services face routine challenges from funders, policy makers, religious organizations, and neighborhood groups that stem from deep-rooted structural stigma against PWUD and community level overdose-related compassion fatigue (Kennedy-Hendricks et al., 2016, 2017; Winstanley, 2020; Yang et al., 2017). These challenges have rippling impacts on staffing, staff burnout, and program implementation, ultimately harming programs’ effectiveness (Akiba et al., 2024).

Research on structural stigma and health inequities has grown over the last decade, including how societal institutions, moral frameworks, and social disorder policing create a system of stigma that alienates PWUD and limits their access to harm reduction services (Friedman et al., 2022; Greene et al., 2022; Hatzenbuehler, 2016). Tsai et al. (2019) explain that public stigma is fueled from ‘stereotypes about PWUD such as their perceived dangerousness or perceived moral failings, which translate into negative attitudes’ toward them. While stigmatization of PWUD is widely documented, there is less discussion around the origins of such stigma (Livingston et al., 2012). Friedman et al. (2022) describe how society’s wealthy and powerful attempt to maintain their status through scapegoating, by causing division in some subsets of the population. The enactment of drug laws and their discriminatory implementation as part of the War on Drugs exemplify scapegoating and social control through political and economic gains for the powerful, and simultaneous harmful consequences for PWUD (Alexander, 2010; Drucker, 2014).

Theoretical frameworks can be a useful tool for intervention development, measurement, research, and policy to reduce stigma. The Health Stigma and Discrimination Framework (HSDF) describes the process of stigmatization across the socio-ecological spectrum in the context of health (Stangl et al., 2019). The HSDF has been flexibly applied across a range of health conditions such as epilepsy, obesity, HIV, and cancer, representing a relevant tool to examine stigma against PWUD.

Given this context, we conducted a qualitative study of the experiences of CBO staff implementing harm reduction services in San Francisco, California. San Francisco represents a notable site for harm reduction in the US, as local CBOs and PWUD have been at the vanguard of implementing innovative interventions for three decades and local medical institutions have implemented innovative models of low-barrier care (Herring et al., 2021). These efforts included early adoption of SSPs in 1988 (Watters et al., 1994), OEND in 1998 (Enteen et al., 2010), FTS distribution in 2018 (Oh et al., 2020), and both community FTIR (Ondocsin et al., 2023) and a sobering center in 2022 (“Drug sobering center opens to serve San Francisco’s SoMa, Tenderloin,” 2022). Our study set out to answer the research question: what are CBOs’ experiences regarding adoption, implementation, and sustainability of harm reduction services in San Francisco?

## 2. Material and methods

### 2.1. Study population

This study is part of a larger evaluation of harm reduction services in San Francisco (R01DA057613). Between July and September 2023, authors AHK, CEM, CFA, LDW, and TM conducted 27 semi-structured interviews with 27 staff sampled from ten CBOs that provided harm reduction services in San Francisco. To our best knowledge, the ten CBOs represented the only organizations providing harm reduction services in San Francisco at the time. Organizations provided one or more of the services outlined in Table 1. We purposively sampled one to three staff at each agency, at distinctly different levels (e.g., leadership, management, frontline workers), to better understand multi-level drivers of adoption, implementation, and sustainability (Palinkas et al., 2015). All ten CBOs were also represented on the Community Advisory Board (CAB) associated with the study, a minority of interviews were done with CAB members themselves (9 of the 27), and CAB members assisted with recruitment of remaining interviewees in-line with CAB best practices (Newman et al., 2011).

We developed an open-ended interview guide that allowed for conversational flexibility around identification of barriers and facilitators to harm reduction intervention adoption, implementation, and sustainability, while maintaining a level of consistency across interviews. Interviews were 45–60 min, conducted either in-person or via video conferencing, audio recorded, and transcribed verbatim. Participants were remunerated $30 for their time. The Institutional Review Board at RTI International approved all study procedures (00022442).

### 2.2. Reflexivity and positionality

Personal reflexivity focuses on researchers’ self-examination of their identities, perspectives, and biases and the related impact on their research (Wilkinson, 1988). Given our study’s inquiry into the experience of implementing harm reduction services, we include this statement with the goal of describing how our team’s positionality, assumptions, and experiences impacted data collection and analysis.

Our analytic team is an interdisciplinary team of epidemiologists, implementation scientists, medical doctors, service providers, and social workers who have a range of 3–30 years of experience in harm reduction research. Our team is diverse and includes people with lived drug use experience.

Based on our experience working with harm reduction organizations, the team brought an understanding of longstanding challenges related to program operations, staffing, and histories. Throughout interviews, we utilized our experiences to probe for additional salient information outside the bounds of our interview guide. The team’s experience doubles as a limitation in that we may hold biases about the barriers and facilitators to program adoption, implementation, and sustainability of services. However, these potential biases were tempered during the analysis phase through regular team discussions where we checked our individual assumptions across the group of researchers. Additionally, we reported preliminary findings back to the study’s CAB members to verify the accuracy and enhance the trustworthiness of our findings. These discussions informed our analysis and ultimately, more nuanced research questions described below.

### 2.3. Data analysis

We used Reflexive Thematic Analysis (RTA) to analyze the interview data due to its flexibility regarding inductive and deductive approaches to coding and generating themes (Braun & Clarke, 2006; Clarke & Braun, 2017). To become familiar with the dataset, we listened to interview recordings without taking notes. This process illuminated stigma against PWUD as a prominent topic of discussion among interviewees. Accordingly, we created an additional research question to incorporate stigma specifically: how does stigma against PWUD or people who provide harm reduction services affect adoption, implementation, and sustainability of harm reduction programs? After listening to all the interviews, we created a codebook with prescribed codes that paralleled the prominent topics that arose in the interviews. At this stage, we chose to incorporate a stigma framework into our codebook.

We used NVivo 12.0 (Lumivero, Denver, CA, USA) as a qualitative analysis tool. In addition to coding, we utilized memo writing to add context to our coding and ultimately build a thematic understanding of our data (Saldaña, 2009). The team held weekly debriefings to review coding and memo writing, add or change existing codes, resolve coding inconsistencies, and discuss the most salient codes and emergence of potential themes. After coding the data, we generated a report on each code. We then grouped code reports into potential thematic categories and annotated each code and category with information about the connections between them. Through the process of “code weaving,” we incorporated our memos into our code report annotations, providing additional support towards thematic development (Saldaña, 2009). After combining memos into our annotated and categorized code reports, the team met to discuss and finalize the themes presented below.

### 2.4. Guiding conceptual models and frameworks

We utilized the HSDF and Proctor’s implementation outcomes within our codebook (Proctor et al., 2011; E. K. Proctor et al., 2009; Stangl et al., 2019). The structured framing allowed us to consider health stigma at different levels and its influence on the implementation of harm reduction services while coding.

The HSDF describes the process of stigmatization across the socio-ecological spectrum in the context of health. The process involves a series of domains, including drivers and multipliers, stigma marking, stigma manifestations, and outcomes. Drivers are inherently negative factors that propagate health stigma; multipliers can be positive or negative influences that attenuate or amplify health stigma. Their combination leads to the occurrence of stigma marking, or the application of stigma to specific groups. This stigma application then manifests in stigma experiences (e.g., discrimination) and stigma practices in the form of beliefs, attitudes, and actions. Ultimately, drivers, multipliers, stigma marking, and stigma manifestations work together to influence a range of implementation and health outcomes for affected populations, organizations, and societies (Stangl et al., 2019).

The listening phase of analysis also highlighted the impact of stigma related challenges on program implementation outcomes like reach, fidelity, feasibility, and sustainability. Accordingly, we incorporated Proctor et al.’s (2009) implementation outcomes into our coding. Implementation outcomes of the degree to which an evidence-based intervention is successfully implemented (Mettert et al., 2020; Proctor et al., 2009).

## 3. Results

**Fig. 1.**
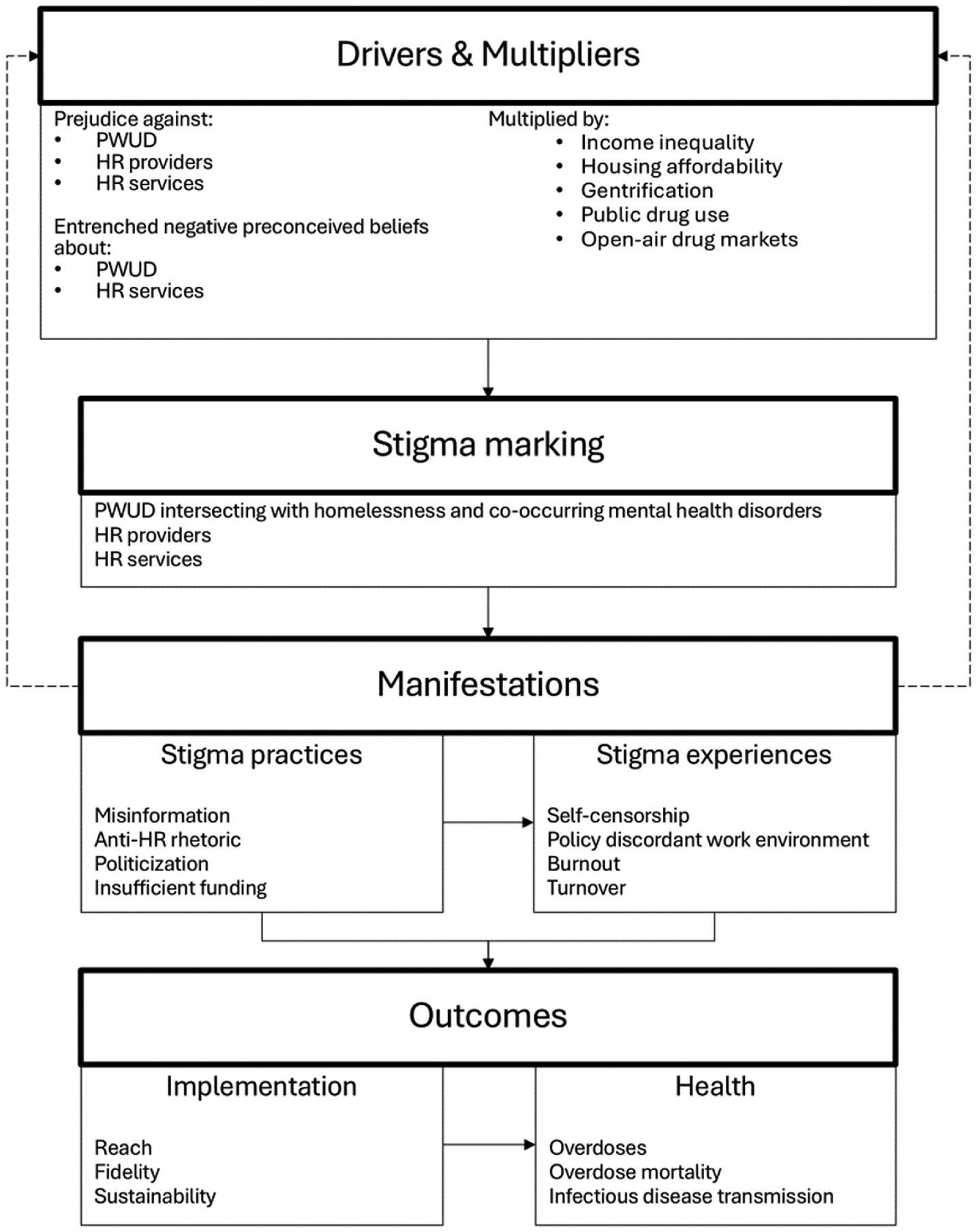
Health stigma and discrimination framework (HSDF) Adapted to present study.

Throughout this section, we present our thematic results by HSDF component (Fig. 1).

### 3.1. Stigma drivers, multipliers, and marking

Interviewees described stigma drivers such as political and community level prejudice against PWUD, harm reduction service providers, and harm reduction services themselves. Several participants perceived elected officials and community members to hold entrenched negative beliefs about PWUD and harm reduction services. One interviewee described the relationship between negative stereotypes that drove neighborhood opposition or NIMBYism (“not in my back yard”) to their organization’s recently implemented sobering center for PWUD and the program’s public funders.

[Our organization] picked a spot [for our new sobering center] then immediately there was a problem … you’ve already got hate sort of fermenting in the neighborhood here, this sort of NIMBYism is kind of enlivened in San Francisco. There’s just been a sort of diminishment in compassion for others, and a reassertion of individuality. “I don’t want it; therefore, it shouldn’t be there …” So that was almost a separate project, managing public negativity. And then we had the Mayor’s Office and the Department of Public Health trying to consolidate their messaging and not just caving into NIMBYism like putting restrictions on our program because an unqualified neighbor has an opinion about PWUD that’s based on negative stereotypes and negative portrayals in the media. So [initially] we had to negotiate a lot of that and now, basically a year in, the NIMBY thing really kind of [settled down], they’re NIMBYing about other stuff because they didn’t get any traction here. [Our sobering center] is doing pretty much everything that we stated, which is taking people off the streets and connecting people to other services. No surprise to anybody who runs [harm reduction] programs but, the NIMBY vision is always, *always*, this exaggerated catastrophic evolution in civility, you know? The rise of criminality and the apocalypse happens right there. And generally, it just has never happened to anyone but it’s almost like that’s the playbook to try and prevent [harm reduction services], you know?

[Leader]

Interviewees also described similar negative beliefs from individuals within their organizations, neighborhood social media accounts, and directly from community members.

Interviewees then described socio-economic multipliers that exacerbated stigma against PWUD or people who provide harm reduction services like extreme income inequality, lack of housing affordability, and gentrification increased public drug use, open-air drug markets, and homelessness. Interviewees described how frustrations grew among community members and politicians city-wide, scapegoating harm reduction as a cause of the social challenges.

I mean, look at what’s going on in this country, [opposition to harm reduction is] just part of that. We have such division, and we have such inequities, and extreme poverty. And no one wants to address [that] or take responsibility for the level of displacement that massive gentrification from the tech industry caused in San Francisco, that’s what this is about … Public health doesn’t matter, everything’s political.

[Leader]

At this point [funding for harm reduction services is] very tenuous because of the politics, because this city has become the third in the nation with the most economic divide. And therefore, there are open air drug markets, there are needles, where even [drug] treatment, including MAT treatment is challenged … so it’s a big narrative, and the majority of people who have moved to San Francisco aren’t okay with that narrative, so it’s very dangerous, we’re in a very dangerous moment I think.

[Leader]

The confluence of stigma drivers (e.g., prejudice and entrenched negative stereotypes) and multipliers (e.g., political-economic conditions) created a landscape in which widespread multi-level opposition to harm reduction could grow.

I think in this city, the group that experiences the most discrimination, demonization, disrespect, and who are just out and out treated kind of like shit and stigmatized in general, are PWUD, especially if they’re experiencing homelessness, and/or have a co-occurring mental health disorder, and are symptomatic, and/or inject … Even [healthcare] providers who are on the right page about so many things, some of those folks balk all of a sudden when a person comes in who uses drugs. Some things that you would never have thought them to say, you’ll hear them say, you know? This is a group that experiences discrimination even from the very last folks I would expect to discriminate, or stigmatize, or prejudge, and finger wag, and preach, and make assumptions, and presumptions, you know? [It’s] totally inappropriate, and if they did it about any other marginalized group there would be a resounding response, right? I think this group is treated in ways that very much could not go down with any other group.

[Frontline staff]

We’re in this political climate where we are hated, like where you’ll see things on Twitter that say, “harm reduction is assisted suicide,” like I myself, at a mobile site last summer, was assaulted by a social media influencer … He pinned me against a table screaming that I “give out killing kits” and “spread disease.”

[Leadership]

Ten years ago we lost our drop-in center, which is where everything ran out of, and we have had a very difficult time getting a new space. Ten years later, and we still don’t have one. So, you know, it’s a bunch of [expletive] [expletive] really. Anyhow, we’ve been under police surveillance, [a high-ranking public official] has tried to shut us down many times, so I’d almost say we’re less stable than we were even ten years ago because of poor political will in this city, because [a high ranking public official] personally hates me and has made it very difficult for us to exist, so. We currently operate syringe services out of [a vacant office space], which has been empty for four years.

[Leader]

We [provide syringe services] in the alley and some drivers, they [expletive] speed through. So we’re always trying to be mindful of people walking in the street because we’re very concerned that someone’s going to do a hit and run. Especially with the hostilities towards PWUD, I’m afraid that someone’s going to run through the alley and try to kill people.

[Leader]

The hate and hostility described by interviewees for PWUD, harm reduction service providers, or for organizations implementing harm reduction programs, highlights their marking as stigmatized groups at the individual, provider, and organizational levels.

### 3.2. Stigma manifestations, outcomes, and solutions

Interviewees described stigma manifestations like anti-harm reduction misinformation and political rhetoric, and harm reduction’s politicization. This created an environment characterized by insufficient public funding for harm reduction programs, harm reduction provider self-censorship, a policy discordant work environment, and staff burnout and turnover. Manifestations harmed downstream program implementation outcomes (e.g., reach, fidelity, sustainability) and ultimately health outcomes for PWUD and staff who provide harm reduction services. Interviewees also described their organizations’ adaptations to overcome stigma-related challenges and suggested solutions to address them.

#### 3.2.1. Misinformation, public funding, and program fidelity

One interviewee described an organized anti-harm reduction group’s spread of misinformation and its intended impact on public funding for harm reduction programs.

The opposition is a very well-funded sort of astroturfey group trying to move San Francisco from a progressive, left-leaning, liberal city to a more centrist business and police friendly city. They are actively trying to take funding away from harm reduction because they say, “it doesn’t work, look at the state of people on the street,” and rightly so, the people on the street are in terrible condition post-COVID. And so, they have joined forces with some of these abstinence only programs … because [those programs] can’t get funded through DPH because they’re not actually certified treatment programs … So, in the end, this is about funding and money … this opposition is putting out a lot of misinformation about harm reduction, that harm reduction “doesn’t want people to get sober,” that harm reduction “just wants people to keep using” … that we’re “enabling drug use” and “not encouraging people to go to treatment.”

[Leadership]

Other interviewees described the spread of harm reduction misinformation through several popular social media influencers and through traditional news media. Interviewees also described an awareness that not only are PWUD and harm reduction services marked with stigma, but they also operate within an environment punctuated by competition for limited public funds. Interviewees highlighted how those conditions created a sense that their organizations needed to implement harm reduction programs with fidelity and disseminate information about program effectiveness to ultimately retain public funding.

I’d say the one thing that I think is important to acknowledge is that with harm reduction and needle distribution sort of in the bullseye right now, and the political movement towards policing, we want to be really mindful as we [provide syringe services], that we don’t damage what is a very successful program to prevent the spread of disease, right?

[Leader]

However, interviewees also expressed frustrations about the need to demonstrate program effectiveness given the robust evidence base for harm reduction interventions.

Right now what we ask if it’s the first time they’re getting Narcan, or if it’s a refill. If it’s a refill, then we ask [what happened to your last dose], did you use it? Did it get taken? What happened to the Narcan? And if they used it, how many doses did they use? Or how many overdoses did they reverse? And we also collect demographics, so gender and race, ethnicity … I think my question is like why are we collecting these data? What is the purpose of it right now? … It feels like a lot of the data are being used politically, to fight against all this anti-harm reduction rhetoric, like “we need the overdose [reversal] numbers to see if [Narcan] is effective or not,” you know? I’m like “it *is* effective, there’s so much data to prove that it is already.” It feels more of a political game than something that’s actually useful.

[Manager]

Despite the focus on data and reporting as tools to convey the impact of harm reduction interventions with previously established efficacy, one interviewee shared that even when they reported on their program’s effectiveness, the information ultimately felt insufficient to garner sustained public funding.

Every conversation that we’ve had with [our public funders], it feels like harm reduction is taboo again. And we had worked so hard to evolve it to be where we were, for it to now be a “bad word” or a “bad phrase” where we can’t even use it in certain reports? Or when we’re talking to specific people? That feels backwards … And I think all of the [harm reduction] programs that we’ve collaborated with also feel that pressure and that tension of doing really impactful work, and completing the reports to actually show that impact … to just be told “now we’re actually moving in a different direction, more abstinence based, we’re giving all our money to the police,” that feels just gross.

[Front-line staff]

Other interviewees also described self-censorship regarding the term “harm reduction,” with one organization choosing to remove the term from a public facing sign, as it drew unwanted community opposition. The impactful work described in the quote above referred to the development and implementation of a community-based intervention that trained residents living in single room occupancy (SRO) housing to reverse overdoses themselves and train fellow residents to do the same. At the time of interview, the public funding supporting this intervention transitioned to a different source, bringing negotiations that resulted in changes to the intervention’s core components.

We also pushed for [the new funding agency] to fund our community-based intervention, which was the intervention for residents because that was really the piece that we found to be more impactful, and they denied us. They were like “we’re just focusing on [training housing] staff,” … but we were really pushing the fact that this is the *actual* community-based intervention with people who are *actually* using drugs in these settings, like we need to fund that intervention. And right now, the political climate in San Francisco is very, very messy but we were told that that wasn’t going to happen as of now … I think no one on the team feels like it’s going to have as much of an impact [on overdoses] as a resident-level intervention.

[Front-line staff]

Due to stigma marking, harm reduction became the target of misinformation, contributing to diminished public and political support. Despite demonstrating effectiveness, interviewees described public funding for harm reduction as inadequate and unstable, with one organization closing its services during the study period due to insufficient funding. The collective challenges harmed program implementation and left some interviewees feeling that their programs were ultimately made less effective.

#### 3.2.2. Anti-harm reduction rhetoric, community opposition, and policy discordance

Interviewees also described how anti-harm reduction rhetoric used by high-ranking public figures contributed to community level prejudice and further engrained negative beliefs about PWUD and harm reduction services (dashed arrows in Fig. 1). In turn, harm reduction became politicized, clearing a path for the enforcement of paradoxically discordant policies that ultimately challenged program implementation.

The politics of the city really influence how the community is perceiving what needle exchanges do and don’t do, and I think [a high-ranking public official] right now is kind of recycling and revamping the War on Drugs and the criminalization of PWUD. I’m noticing on a kind of neighborhood community level, and also within the Board of Supervisors, just in some of their rhetoric, the things they’re choosing to take the time on … it’s all just like this trickle down from what we’re seeing with [that high-ranking public official].

[Manager]

In an environment marked by interwoven political and community opposition to harm reduction services, interviewees described harm reduction’s politicization, with one explaining its impact on the implementation of their organization’s overdose prevention campaign.

We did a poster campaign, it had pictures from the community, they were consensual, it was community based, and PWUD were in the photos. It was about showing tips on overdose prevention and some of the pictures of people looked like they were having fun. We got an e-mail a few months ago because [some city] politicians saw it and started attacking the DPH and suddenly DPH [who fund our program] were like, “you’re not allowed to publish anything without consulting with us, and you cannot use [these posters] anymore.” We had to take it all down in less than a day. And for me that’s a very practical way that funders are limiting what we get to do … what we can actually say … And it’s sometimes concerning that if the politics shift so much in San Francisco, or like the pressures on the leadership that provide us with funding, that we might have to change our language and change what we’re trying to say and how we educate people to fit an anti-harm reduction narrative … it’s scary to see how that is shifting.

[Manager]

The interviewee reflects a fear depicting a public health approach in which politics play an active role in the design of intervention content, rather than harnessing decades of public health evidence and expertise. Some interviewees added that the politicized environment contributed to an ecosystem in which public policies paradoxically constrained harm reduction program implementation, in turn limiting the effectiveness of services, wasting public resources, and demotivating staff.

When the new [high ranking public official] started, we had cops following us on outreach. We would give out our pipes, they’d be right behind us and then they would go up to the people that we just gave pipes to and smash their pipes, which is kind of hilarious because the city is paying us to give out these pipes and then the city is also now paying to destroy them. But that’s not only difficult and makes your job harder, it’s incredibly demoralizing.

[Manager]

The seizure and destruction of safer smoking supplies constrained program reach, or the number of participants that might benefit from a given intervention. Similarly, another interviewee described participants’ hesitancy to engage in their organization’s community FTIR service for fear of arrest due to drug possession. Such fears harmed FTIR feasibility, or ease of use, which requires participants to present drug samples to test for potentially life-threatening adulterants.

Altogether, politicians’ use of anti-harm reduction rhetoric seemed to further engrain stigma drivers and promote community opposition. The combination created a largely anti-harm reduction setting in which politicization and the enforcement of paradoxical policies worked to challenge the implementation of harm reduction interventions.

#### 3.2.3. Insufficient budgets, staffing challenges, and program implementation

When asked about program funding, interviewees from nearly every organization described their programs’ budgets as insufficient. Due to increasing poverty and homelessness among PWUD in San Francisco, staff also described their organizations’ efforts to provide additional survival services like shelter, hot meals, and medical care. While essential for program participants, the additional services further taxed organizations’ already strained budgets and staffing capacities. Staff also described additional challenges related to workplace trauma. The constellation of funding and staffing challenges led to burnout, turnover, and insufficient staffing, ultimately threatening program reach, fidelity, and sustainability.

Harm reduction programs still operate on a shoestring budget compared to all the other money that goes into the city to support the DPH’s [programs]. So that’s a reality. And in fact … no funding allocation increases have happened for harm reduction programs. We’re now having to fight a narrative [that harm reduction is ineffective], and also, we have to fight for survival.

[Leader]

In addition to funding challenges, interviewees described staff trauma related to politicians’ stigmatizing and anti-harm reduction rhetoric, harm reduction’s politicization, and related community opposition. They mentioned feeling “hated,” “incredibly demoralized,” “heartbroken by the lack of political support for harm reduction services,” “[getting] spit on by anti-harm reduction folks,” regular confrontations with people who “yell at us or blame us for what’s going on,” and concerningly, one who described being assaulted by a social media influencer, leading their organization to hire security guards to ensure staff safety.

You’re getting assaulted at work … You’re experiencing *primary trauma*. You’re *in* the traumatic event. You’re not hearing about it from a client telling you about a bad thing that happened to them … How do you retain staff? … It feels like you have to operate your site like you’re Planned Parenthood. We’ve upped it now to two security guards.

[Leader]

Challenges related to staff trauma became compounded as interviewees described how insufficient funding led to inadequate staff wages. In a callback to the city’s housing affordability crisis, interviewees described burnout and turnover related to inadequate compensation accompanied by an inability to comfortably live where they work. One interviewee noted that their lengthy commute reduced the amount of time available to decompress, further contributing to feelings of burnout and job-related stress. Interviewees highlighted turnover as the endpoint of burnout, trauma, and stress, ultimately harming program implementation.

When staffing levels drop, we can’t do foot outreach. We have to have this hierarchy of what absolutely has to get done, and anytime we reduce staff, we have to figure out where we’re going to pull from.

[Frontline staff]

Insufficient funding for harm reduction programs combined with the trauma of providing stigmatized services severely impacted staff well-being and contributed to burnout and turnover. Staffing challenges ultimately limited the number of participants programs were able to serve (reach), their ability to implement harm-reduction programs as intended (fidelity), and their ability to adequately maintain harm reduction programming (sustainability).

#### 3.2.4. Organization level mutual aid, political support, and program implementation

When asked about activities that supported the adoption, implementation, and sustainability of harm reduction services, interviewees from every organization described a network of formal and informal mutual aid among local harm reduction organizations that included sharing supplies, staff, or services.

We got linked up with a gentleman who works for [a local university] and he’s provided us with a much more official and efficient system for getting harm reduction supplies. So basically, he gives us an order form, we put in what we need, and within a few days they bring it all out.

[Manager]

When asked about how they first connected with the local university, the interviewee described an intentional effort to seek out mutual aid at the organizational level.

Our Director of Operations spends most of their time attending the monthly local harm reduction meetings. Basically, I would say 90 % of their time is spent building and seeking out relationships with partner organizations.

[Manager]

This focus on relationship building suggests the importance of mutual aid at the organizational level.

Interviewees from one organization also highlighted the impact of a member of the Board of Supervisors in facilitating the adoption of community FTIR services. They described how the Supervisor approached them with an offer to fund an innovative harm reduction program to address overdose deaths in San Francisco.

At the end of the year the District Supervisor [told us there was] extra money in the budget, [the Supervisor] met with us and asked what else could we be doing? What out of the box thinking is there around overdose prevention? And myself and [leaders of my organization] pitched community FTIR and he liked it and gave us money to buy the first drug checking machine.

[Leader]

The interviewee highlights how the engagement of a single politician facilitated the adoption of community FTIR services. The machine’s cost (US$23,391) represented a barrier that might otherwise prohibit the organization’s adoption. The Board member’s desire to engage with a harm reduction organization stands in contrast with most interviewees’ depictions of local politicians. Most staff described feeling unsupported, and some described feeling “hated.” While interviewees described benefiting from organizational mutual aid in terms of program adoption, reach, and sustainability, nearly all still characterized their programs as insufficiently funded and reflected overall fears regarding program sustainability.

## 4. Discussion

Interviewees illustrated how they, as providers of harm reduction services, and harm reduction services themselves, experienced various forms of stigma at the provider, community, and policy level. These results align with extant literature regarding the stigmatization of harm reduction services and their provision (Brener et al., 2024; Davis et al., 2023; Glover & Sporer, 2025; Pasman et al.; B. White et al., 2016).

Interviewees acknowledged how political, economic, and social forces like the lack of housing affordability, gentrification, and tech industry job loss in the wake of the COVID-19 pandemic contributed to the rise of extreme income inequality, homelessness, public drug use, open air drug markets, and unintentional drug overdoses in San Francisco. City and industry figures confirm that the unhoused population, income inequality, tech industry layoffs, and overdose deaths grew to all-time highs during the post-pandemic period (“Drug overdose and treatment data and reports,” 2024; “Homeless Population,” 2024; “How has income inequality changed in the Bay Area over the last decade?,” 2021; “U.S. Census Bureau, Income Inequality in San Francisco County, CA ”). These social determinants and their impact on drug use and overdose mortality are not unique to San Francisco. In their Perspectives piece published by the American Journal of Public Health, Dasgupta et al. (2018) describe how “structural factors such as poverty, lack of opportunity, and substandard living and working conditions” impact health outcomes generally, and overdose mortality in particular. In their nationwide study of public drug use, Sutter et al. (2019) reported independent and significant associations between increased odds of frequent public drug use and street homelessness, unstable housing, and being under age 30 respectively. While not discussed by our interviewees, Winstanley’s (2020) commentary on overdose-related compassion fatigue may represent another potential contributing factor to the stigmatization of harm reduction in San Francisco. Winstanley (2020) defines the term as “distress or a reduction in empathy resulting from knowledge of or exposure(s) to overdose-related harms” and describes how, when experienced at the community level, overdose-related compassion fatigue can result in diminished support for harm reduction services while breathing life into stigmatizing and punitive drug policies. Future research examining overdose-related compassion fatigue among community members and business owners may facilitate a greater understanding of the multitude of factors that impact community support for harm reduction.

Despite complex political, economic, and social determinants, interviewees described the spread of anti-harm reduction misinformation that blamed harm reduction for the city’s structural challenges. The spread of misinformation on social media represents a serious public health threat given that majority of Americans receive health information online (Fox, 2011). Misinformation can be corrected through counter-messaging (Fox, 2011; Smailhodzic et al., 2016). In their meta-analysis of communication strategies that combat misinformation on social media, Walter et al. (2020) found that posts correcting misinformation were most effective when those posting were involved in the health topic, and when misinformation was distributed by news organizations (rather than peers) and discredited by experts (rather than non-experts) (Walter et al., 2021). Future research should examine the most effective counter messages for anti-harm reduction misinformation online.

Friedman and colleagues’ (2022) Stigma System describes how stigma can act as a tool to achieve and maintain power. They highlight how those in power partition populations into stigmatized out-groups and non-stigmatized in-groups, where out-groups become scapegoated as the cause of social challenges to build multi-level support among the in-group and ultimately maintain power (Friedman et al., 2022). Similarly, in creating typologies of stigma related to opioid use disorder, Tsai et al. (2019) highlight how forms of stigma run at cross purposes to reduce public support for public health–oriented policies that address the opioid overdose crisis (Tsai et al., 2019). The findings of Friedman et al. (2022) and Tsai et al. (2019) resonate with our results where interviewees felt the decades-long literature base regarding harm reduction efficacy was ultimately insufficient to garner public support amid anti-harm reduction misinformation, public rhetoric, and politicization.

In their work with national and state-level harm reduction advocates on communication strategies to reduce stigma, White et al. (2023) describe how the most effective strategies (1) matched harm reduction and audience-specific values and (2) framed harm reduction as one part of a constellation of strategies to address the overdose epidemic. The authors also highlight the importance of tailoring messages to key audiences like policymakers, law enforcement, religious groups, and family and friends of PWUD (White et al., 2023). Services for similarly stigmatized health conditions like HIV, mental health disorders, and disabilities have seen reductions in community stigma via support from public figures (Barry & McGinty, 2014; Hatzenbuehler et al., 2020; Rao et al., 2019; Wang & Ashburn-Nardo, 2019).

Study participants described engaging in organizational-level mutual aid and sharing resources to facilitate program adoption, reach, and suitability. This phenomenon may represent the confluence of organizational behavior among non-profits with shared goals, and the culture of harm reduction. In their study of collaboration typologies among non-profit organizations, Rathi et al. (2014) describe Sector Partnerships in which non-profits from similar sectors work together to share scarce resources (Rathi et al., 2014). Harm reduction also aligns with a culture of mutual aid, in their qualitative study of mutual aid and altruism in harm reduction, Bathje and colleagues (2020) describe how harm reduction tends to utilize mutual aid among PWUD to counteract drug user stigma (Bathje et al., 2020). Organizations in our study used mutual aid as a stop gap measure, mitigating implementation barriers. Nevertheless, most programs ultimately reported pervasive challenges regarding program adoption, implementation, and sustainability.

Because our sample focused on organizations providing harm reduction services within San Francisco, our inference remains restricted within the city. We conducted our study after a period of the COVID-19 pandemic during which the city invested in the expansion of harm reduction interventions, including a city-funded overdose prevention site (Suen et al., 2023). As interviewees described, the level of homelessness, public drug use, and overdose deaths persisted within the city, resulting in public frustration and ultimately the scapegoating of harm reduction services described throughout this manuscript. During the lead-up to the 2024 mayoral election, then Mayor London Breed who previously voiced support for harm reduction, even lobbying the Biden administration to expand federal funds for harm reduction interventions exemplified this shift by asserting that “harm reduction, from my perspective, is not reducing the harm … it is making things worse (Greschler, 2024; Mann, 2022; “Mayor London Breed Leads Resolution Calling for Federal Action on Fentanyl,” 2023).” Similarly, in 2023 Mayor Jim Kenney of Philadelphia invested a portion of the city’s $200 million opioid settlement funds into organizations providing harm reduction services and voiced support for an overdose prevention site (Leonard, 2023a, 2023b; Peterson, 2023). However, after winning the 2024 Philadelphia mayoral race, Cherelle Parker vowed that “not one city dollar” would be used to fund needle exchange programs in their distribution of drug use supplies (Orso & Whelan, 2024). At the state level, starting in 2017 the Idaho Department of Health and Welfare provided naloxone to a range of public and non-profit organizations for free, including harm reduction groups, to curb rising overdose deaths (Suppe, 2023). In 2023, the state legislature passed a bill constraining the use of state funds earmarked for syringes and naloxone to first responders only, despite data showing that first responders accounted for only 6 % of overdose reversals statewide during the prior year. In West Virginia, the 2024 legislative session saw the passage of HB 4667 that outlawed SSPs from distributing safe smoking supplies, a move that mirrored the Biden administration’s 2022 restrictions on the use of federal harm reduction funds for safe smoking supplies (Alonso-Zaldivar & Dupuy, 2022; Coyne, 2024). While we cannot comment on contributing factors behind these legislative and executive actions, they seem to mirror the arc described by our interviewees in San Francisco, where initial city, state, or federal investments in harm reduction were followed by swift rebukes.

Our work exhibited several strengths including the flexibility inherent in RTA as well as our utilization of the HSDF to facilitate our understanding of multi-level stigma and its determinants. RTA’s initial data familiarization step of listening to interview recordings illuminated stigma as a topic of interest. This step allowed us to inductively code for stigma and its determinants. It also necessitated the need for a structured understanding of stigma, leading us to the HSDF. The HSDF helped situate stigma against harm reduction at multiple levels, clarify the pathways between them, and illuminate potential stigma-reduction interventions.

At the same time our findings should be understood within the presence of several potential limitations. For example, the harm reduction organizations in this sample stemmed from our knowledge of existing programs. Because of harm reduction stigma, programs commonly operate underground to avoid law enforcement or community harassment (Smith, 2012; Wieloch, 2002). It remains possible that additional harm reduction organizations existed within San Francisco during the study period whose voices are not included in our work. Our future work will harness the expertise of our CAB to determine additional programs for study inclusion. Because our original research question did not focus on stigma explicitly, we were unable to fully explore its impacts and determinants. To that end, future data collection efforts will focus on harm reduction stigma explicitly.

Stigma against PWUD, harm reduction providers, and harm reduction services themselves, combined with political, social, and economic challenges, ushered in a cascade of impediments to program implementation for organizations in our study. Targeting stigma directly may go far in addressing those impediments over the long term but additional, adequate, and sustained funding are needed to address short term challenges. In lieu of that, programs will likely continue to rely on a system of organizational mutual aid however fragile it might be, within an ecosystem of authoritarian and punitive drug policy.

## Figures and Tables

**Table 1:** Harm reduction interventions - Descriptions, associated outcomes, and evidence-base.

Intervention	Description	Associated Outcomes	Evidence-Base
Syringe services programs (SSP)	Distribute safer drug use supplies, like sterile syringes and smoking equipment Offer other evidence-based interventions including wound care, infectious disease testing and treatment, and linkages treatment	Viral and bacterial disease transmission Overdoses, overdose mortality Engagement in care Hospital/emergency room utilization	(Bartholomew, Tookes, Bullock et al., 2020; Bluthenthal, Anderson, Flynn, & Kral, 2007; Bluthenthal, Kral, Gee, Erringer, & Edlin, 2000; Bluthenthal, Kral, Lorvick, & Watters, 1997; D. C. Des Jarlais, Arasteh et al., 2009; Kral, Anderson, Flynn, & Bluthenthal, 2004; Watters et al., 1994)
Overdose education and naloxone distribution (OEND)	Educate people on how to respond to an overdose event and administer naloxone Implemented within SSPs and other CBOs	Overdoses, overdose mortality	(Doe-Simkins et al., 2014; Doe-Simkins, Walley, Epstein, & Moyer, 2009; Jones et al., 2021; Lambdin, Davis, Wheeler, Tueller, & Kral, 2018; Walley et al., 2013)
Overdose prevention sites (OPS)	Settings where drug use is supervised by trained staff who intervene in the event of an overdose or other health complications. Also provide linkage to myriad social and health services.	Viral and bacterial disease transmission Overdoses, overdose mortality Engagement in care Hospital/emergency room utilization	(Samuels, Bailer, & Yolken, 2022; Wood et al., 2006; Kral et al., 2021; Lambdin et al., 2022; Suen et al., 2022; Davidson et al., 2023; Giglio et al., 2023; Harocopos et al., 2022; Suen et al., 2022; Suen et al., 2023; Wenger et al., 2024)
Community-based Fourier-transform infrared spectroscopy drug checking (FTIR)	Machine based testing of drug samples to identify substances present and the ratio of the sample’s components	Overdoses, overdose mortality	(Karch et al., 2021; Tupper, McCrae, Garber, Lysyshyn, & Wood, 2018)
Fentanyl test strips (FTS)	Point-of-care tests that allow people to test their drugs for fentanyl	Overdoses, overdose mortality	(Park et al., 2021; Peiper et al., 2019; Zibbell et al., 2024)
Overdose detection technologies (Brave Buttons)	Brave Buttons are designed for supportive housing, emergency shelters, and complex care environments to keep residents safe by allowing them to contact staff through smart phone applications, hotlines, and wearable technology when they need overdose-related care	Overdoses, overdose mortality	(Bardwell et al., 2021; Goldfine et al., 2020; Lombardi et al., 2023; Nuamah et al., 2020; Welwean et al., 2023)
Drug and alcohol sobering centers	Places where individuals who are under the influence of drugs and/or alcohol can stay, to rest, remain safe, and to be linked with additional resources	Overdoses, overdose mortality Engagement in care Hospital/emergency room utilization Mental health concerns (e. g., psychosis)	(Jarvis et al., 2019; Marshall et al., 2021; Smith-Bernardin, 2021; Smith-Bernardin et al., 2017)

## Abbreviations:

CAB: Community Advisory Board
CBO: Community-Based Organization
FTIR: Fourier Transform Infrared Spectroscopy
FTS: Fentanyl Test Strips
HSDF: Health Stigma and Discrimination Framework
OEND: Overdose Education and Naloxone Distribution
OPS: Overdose Prevention Site
PWUD: People Who Use Drugs
RTA: Reflexive Thematic Analysis
SSP: Syringe Services Program
